# Einsatz und Nutzen von Gesundheits-Apps in chirurgischen Disziplinen – ein Delphi-Experten-Konsensus

**DOI:** 10.1007/s00104-025-02329-5

**Published:** 2025-07-07

**Authors:** C. Groeben, P. Karschuck, F. Kormann, M. Baunacke, L. Wiemer, H. Krause, K. Fuchs, A. Wiedemann, A. A. Schnitzbauer, A. Schmitz, D. Ebert, J. P. Struck, H. Borgmann

**Affiliations:** 1 Klinik für Urologie, Philipps-Universität Marburg, Baldingerstr., 35033 Marburg, Deutschland; 2 Klinik und Poliklinik für Urologie, Technische Universität Dresden, Dresden, Deutschland Fetscherstrasse 74, 01307; 3 Kranus Health GmbH, Berlin, Deutschland 10178, Karl-Marx-Allee 3,; 4 Klinik für Urologie und Kinderurologie, Charité Universitätsmedizin, Berlin, Deutschland Charitéplatz 1, 10117; 5 INSPIRE Living Lab, Universitätsklinikum Mannheim, Mannheim, Deutschland Theodor-Kutzer-Ufer 1-3, 68167; 6 Klinik und Poliklinik für Unfall‑, Hand‑, Plastische und Wiederherstellungschirurgie, Universitätsklinik Würzburg, Würzburg, Deutschland Oberdürrbacher Strasse 6, 97080; 7 Urologische Abteilung, Evangelisches Krankenhaus Witten gGmbH, Lehrstuhl für Geriatrie, Universität Witten/Herdecke, Witten, Deutschland Pferdebachstrasse 27, 58455; 8 Chirurgische Klinik, Universitätsklinikum Knappschaftkliniken Bochum, Ruhr Universität Bochum, Bochum, Deutschland Universitätsstraße 150, 44801; 9 Research & Development, Pharmaceuticals, Digital Health & Real-World-Science, Data Science & AI, Bayer AG, Berlin, Deutschland Müllerstrasse 178, 13353; 10 Professur für Psychology & Digital Mental Health Care, School of Medicine & Health, Technische Universität München, München, Deutschland Georg Brauchle Ring 60, 80992; 11 Klinik für Urologie und Kinderurologie, Universitätsklinikum Brandenburg an der Havel, Brandenburg, Deutschland Hochstrasse 29, 14770; 12 Urologische Universitätsklinik Heidelberg, Medizinische Fakultät, Universität Heidelberg, Heidelberg, Deutschland Im Neuenheimer Feld 420 , 69120

**Keywords:** Digitale Gesundheitsanwendungen (DiGA), Chirurgie, Urologie, Delphi-Verfahren, Patientensupport, Digital health application (DiGA), Surgery, Urology, Delphi procedure, Patient support

## Abstract

**Einleitung:**

Gesundheits-Apps oder digitale Gesundheitsanwendungen (DiGA) bieten großes Potenzial für die Modernisierung der Gesundheitsversorgung, stellen jedoch hohe Anforderungen an die digitale Gesundheitslandschaft. Um einen reellen Mehrwert für die Patientenversorgung zu schaffen, müssen klare Kriterien erfüllt sein. Diese Arbeit basiert auf einer Konsensuskonferenz des Digital Health Summits (29./30.08.2024) an der Technischen Hochschule Brandenburg.

**Material und Methodik:**

Ein modifiziertes, mehrstufiges Delphi-Befragungsverfahren wurde mit interdisziplinären Experten aus Klinik, Wissenschaft und Industrie durchgeführt, um Konsens zu Anforderungen an chirurgische Gesundheits-Apps zu erzielen.

**Ergebnisse:**

Das Delphi-Verfahren führte zu 30 Statements über Anforderungen an chirurgische Gesundheits-Apps in Deutschland. Sie können evidenzbasierten Nutzen bei der Patienteninformation und Symptomreduktion bieten, müssen jedoch verstärkt in klinischen Studien auf Nutzen und Sicherheit geprüft werden. Apps können zudem die Ausbildung unterstützen, Dokumentation vereinfachen und Prozesse effizienter gestalten. Einheitliche Qualitätskriterien im europäischen Kontext sind nötig. Patientendaten sollten anonymisiert der Forschung dienen, wobei die Datensouveränität bei den Patient*innen liegt. Regulatorische Hürden sollten abgebaut und DiGAs nach Evidenz und Risiko-Nutzen-Profil klassifiziert werden.

**Schlussfolgerung:**

In unseren Statements empfehlen wir, die Entwicklung und Nutzung von Gesundheits-Apps aktiv zu fördern, um die Patientenversorgung in der Chirurgie zu verbessern. Dies erfordert gezielte Unterstützung für Zulassung, Forschung und Nutzung, besonders durch akademische Gruppen, sowie Studien zur Effektivität von Gesundheits-Apps.

**Graphic abstract:**

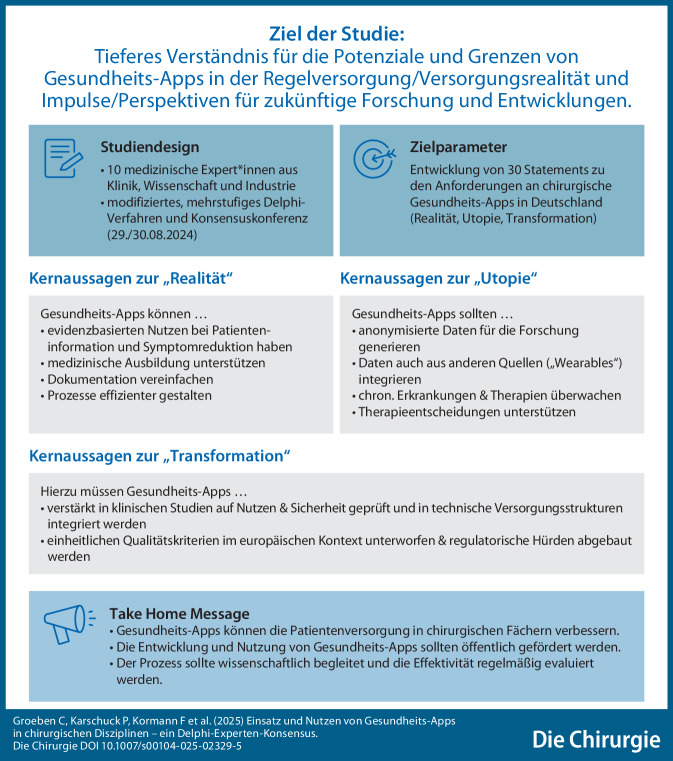

**Zusatzmaterial online:**

Die Online-Version dieses Artikels (10.1007/s00104-025-02329-5) enthält eine zusätzliche Tabelle.

## Abstract (en)

**Background:**

Healthcare apps or healthcare applications (DiHA) offer great potential for the modernization of healthcare treatment but place high demands on the digital health landscape. In order to achieve a real additional value for the treatment of patients, clear criteria must be fulfilled. This article is based on a consensus conference of the Digital Health Summit (29–30 August 2024) at the Technical University Brandenburg.

**Material and method:**

A modified multistage Delphi survey procedure was carried out with interdisciplinary experts from clinical, scientific and industrial sectors to achieve a consensus on the requirements for surgical healthcare apps.

**Results:**

The Delphi procedure led to 30 statements on requirements for surgical apps in Germany. They can provide evidence-based benefits for patient information and reduction of symptoms but must be more stringently tested in clinical studies on benefits and safety. In addition, apps can support training, simplify documentation and organize processes more efficiently. Uniform quality criteria are necessary in the European context. Patient data should be anonymized to assist research, whereby the sovereignty of the data lies by the patients. Regulatory hurdles should be dismantled and DiHAs should be classified according to the evidence and the risk-benefit profile.

**Conclusion:**

In our statements we recommend to actively promote the development and use of healthcare apps to improve the treatment of patients in surgery. This requires targeted support for licensing, research and use, particularly by academic groups, as well as studies on the efficacy of healthcare apps.

In den letzten Jahren hat sich der Gesundheitssektor durch technologische Innovationen rasant weiterentwickelt. Eine der bedeutendsten Entwicklungen ist die Verbreitung von Gesundheits-Apps [1]. Diese mobilen Anwendungen bieten Funktionen wie die Überwachung von Vitaldaten, Vermittlung von Wissen über Erkrankungen, die Verwaltung chronischer Krankheiten und die Förderung gesunder Lebensstile. Durch die Integration in den Alltag der Nutzer eröffnen sie neue Möglichkeiten für die Prävention, Diagnose und Behandlung von Krankheiten [2]. Gesundheits-Apps ermöglichen es Patient*innen, ihre Gesundheit aktiv zu überwachen und zu steuern, was zu einer verbesserten Autonomie und Beteiligung führt [3].

Die Verfügbarkeit von Gesundheits-Apps hat nicht nur die Art und Weise verändert, wie Gesundheitsinformationen gesammelt und genutzt werden, sondern auch die Interaktion zwischen Patient*innen und Gesundheitsdienstleistern [4–7]. Diese Apps können eingegebene Daten ortsunabhängig in Echtzeit verarbeiten, personalisierte Gesundheitsstrategien bieten und zur frühzeitigen Erkennung von Gesundheitsproblemen beitragen. Insbesondere in ländlichen oder unterversorgten Gebieten, in denen der Zugang zu medizinischer Versorgung begrenzt ist, könnten sie von großem Vorteil sein [8].

Seit der ersten Zulassung einer digitalen Gesundheitsanwendung (DiGA) vor 4 Jahren gibt es derzeit 57 verschreibungsfähige Apps [9]. Zwischen Herbst 2020 und September 2023 wurden knapp 375.000 DiGA-Verschreibungen eingelöst [10], was ein erstes positives Fazit zulässt [11, 12]. Dennoch bestehen Herausforderungen, insbesondere in den Bereichen Datenschutz, Datensicherheit und der Genauigkeit der bereitgestellten Informationen. Zudem erfordert die Regulierung von Gesundheits-Apps hohe Anforderungen an Sicherheit, wie z. B. der Kriterienkatalog C5 des Bundesamts für Sicherheit in der Informationstechnik (BSI), was für Hersteller zu hohen Belastungen führt [13].

Die vorliegende Arbeit ist das Ergebnis einer Konsensuskonferenz im Rahmen des Digital Health Summit 2024 in Brandenburg a. d. Havel. Eine Gruppe von Mediziner*innen und Wissenschaftler*innen aus dem Bereich Gesundheits-Apps und DiGAs hat im Rahmen eines Breitband-Delphi-Verfahrens Grundsätze erarbeitet, die in die Themenbereiche Realität, Utopie und Transformation gegliedert sind. Diese Unterteilung beschreibt verschiedene Aspekte von CE(Conformité Européenne)-zertifizierten, als Medizinprodukt zugelassenen Gesundheits-Apps, darunter Funktionalität, Nutzen und Herausforderungen im Hinblick auf den aktuellen Stand der Technik (Realität), potenzielle Entwicklungen (Utopie) und den Weg dorthin (Transformation).

Ziel der Studie war es, Missstände in der Verbreitung und Implementierung der Technologie zu identifizieren und Lösungsansätze zu formulieren. Diese Arbeit soll ein tieferes Verständnis für die Potenziale und Grenzen von Gesundheits-Apps bewirken und Impulse für zukünftige Forschung und Entwicklungen geben.

## Material und Methoden

Im Folgenden werden die nachstehenden Begriffe verwendet:

*Gesundheits-App:* Digitale Anwendung für mobile Endgeräte, die darauf abzielt, die Gesundheit und das Wohlbefinden der Nutzer zu fördern, zu überwachen oder zu verwalten. Sie kann Funktionen zur Erfassung, Analyse und Bereitstellung von Gesundheitsdaten sowie zur Unterstützung bei der Entwicklung gesunder Verhaltensweisen und der Bereitstellung medizinischer und gesundheitsbezogener Informationen umfassen und kann sowohl von Patient*innen allein als auch mit dem behandelnden Arzt genutzt werden.

*DiGA (digitale Gesundheitsanwendung):* Gesundheits-App oder Web-Applikation, die in Deutschland verschreibungsfähig ist und einen Prüfungsprozess durch das BfArM (Bundesinstitut für Arzneimittel und Medizinprodukte) durchlaufen hat. DiGAs sind nicht nur mobil, sondern umfassen auch Web- und hybride Applikationen (Web + Mobil).

Wir führten ein modifiziertes Delphi-Verfahren mit interdisziplinären Forscher*innen zum Thema Gesundheits-Apps in der Chirurgie durch. Das Expert*innenpanel wurde durch Einladungen an in Deutschland tätige Erst‑/Letztautoren von Forschungsarbeiten auf PubMed sowie weitere Experten zusammengestellt. Hierbei wurden insgesamt 10 klinisch tätige Chirurg*innen, nicht-medizinische Wissenschaftler*innen und Vertreter*innen der Industrie gewonnen (Teilnehmer*innen: Supplements Tab. 1). Die Methodik wurde in Zusammenarbeit mit dem Zentrum für Wissenstransfer „UroEvidence“ der Deutschen Gesellschaft für Urologie konzipiert. Die Delphi-Methode ist ein strukturiertes, mehrstufiges Befragungsverfahren zur Konsensbildung, das auf anonymisierten Einschätzungen eines Expert*innenpanels basiert.

Die Methodik des modifizierten Delphi-Verfahrens wurde durch ein schriftliches Protokoll festgelegt. Alle eingeladenen Expert*innen gaben ihre Interessenkonflikte offen an. Den Expert*innen wurde ein Projektexposé mit relevanten Hintergrundinformationen zur Verfügung gestellt. Das Protokoll umfasste auch eine Literaturrecherche zum Einsatz, Nutzen und Potenzial von Gesundheits-Apps in der Chirurgie.

Es fanden 2 Online-Meetings sowie ein abschließendes Hybrid-Meeting (Präsenz + Online) statt. Im ersten Online-Meeting erfolgte die Projektvorstellung und Sammlung von Kernfragen und Argumenten. Anschließend entwickelten die Konsensusgruppen-Teilnehmer*innen nach Literaturrecherche passende Konsensusstatements, die in die Bereiche Realität, Utopie und Transformation kategorisiert wurden. Eine Konsensusumfrage wurde auf der Plattform surveymonkey.com zur Abstimmung an die Expert*innen versandt. Der Grad der Zustimmung/Ablehnung zum Konsensusstatement erfolgte auf einer 5‑Punkt-Likert-Skala.

Im zweiten Online-Meeting wurden die Abstimmungsergebnisse vorgestellt und diskutiert. Die Statements wurden abhängig vom Zustimmungsgrad angepasst oder verworfen. Eine zweite Konsensusumfrage wurde durchgeführt. Beim finalen Hybrid-Meeting wurden die Konsensusstatements abschließend diskutiert, angepasst und abgestimmt.

Der Grad der Konsensusstärke in Abhängigkeit der Zustimmung der Expert*innen zu den Konsensusstatements wurde gemäß dem AWMF(Arbeitsgemeinschaft der Wissenschaftlichen Medizinischen Fachgesellschaften)-Regelwerk Leitlinien als „Konsens“, „mehrheitliche Zustimmung“ und „keine mehrheitliche Zustimmung“ definiert. Zustimmung wurde als Auswahl der Beantwortungsoptionen der Likert-Skala „stimme zu“ oder „stimme voll und ganz zu“ gewertet.

### Ergebnisse der Konsensuskonferenz

Durch die Mitglieder der Konsensusgruppe wurden initial 46 Statements erarbeitet, in insgesamt 3 Abstimmungsrunden eingebracht und in dazwischenliegenden Online-Diskussionsrunden diskutiert. Durch Kombination ähnlicher Aussagen konnte die Anzahl auf 35 in der 2. Runde und abschließend in der 3. Runde auf 30 Statements reduziert werden, die in der finalen Version mit 100 % Konsens angenommen wurden. Die einzelnen Statements werden in Tab. 1 dargestellt.

**Tab. 1:** Delphi-Umfrage Konsensusgruppe Gesundheits-Apps. Zusammenfassung aller Statements (3. Abstimmungsrunde)

Nr.	2. Delphi-Umfrage Konsensusgruppe „Gesundheits-Apps“	Zuordnung
1	Gesundheits-Apps können Symptome und Beschwerden von Krankheiten und krankhaften Zuständen reduzieren. Eine wissenschaftliche Evidenz nach Kriterien der DiGA-Zulassung liegt z. B. für die folgenden urologischen Indikationen vor: erektile Dysfunktion, Endometriose, „overactive bladder“/benigne Prostatahyperplasie, Ejaculatio praecox und Harninkontinenz (ohne Anspruch auf Vollständigkeit)	Anwendung
2	Anwendungsbereiche von in Deutschland derzeit zugelassenen DiGAs umfassen chirurgische, internistische und psychologische/psychiatrische Themengebiete. Den prozentual höchsten Anteil stellen Apps mit dem Schwerpunkt Psychiatrie und Psychotherapie dar	Anwendung
3	Gesundheits-Apps können einen evidenzbasierten Nutzen in der Patienteninformation haben	Anwendung
4	Der Nutzen und die Sicherheit von Gesundheits-Apps sollten/sollen in klinischen Studien überprüft und statistisch begleitet werden	Forschung
5	Gesundheits-Apps sollten Patient*innen unabhängig von deren/dessen Alter angeboten werden	Zielgruppe
6	„Arzt-Apps“ können die ärztliche/chirurgische Ausbildung bei konkreten Tätigkeiten unterstützen und somit zur Verbesserung therapeutischer Anwendungen beitragen	Anwendung
7	Eine feste Integration von Krankenhausarbeitsplatzsystemen auf mobilen Endgeräten könnte den Dokumentationsaufwand und die Prozessabläufe in Gesundheitseinrichtungen effizienter gestalten	Arzt-App
8	DiGAs sollten die Überwachung und Therapie chronischer Erkrankungen unterstützen	Anwendung
9	DiGAs könnten dabei helfen, personalisierte Gesundheitspläne und Empfehlungen basierend auf individuellen Gesundheitsdaten und -verläufen zu erstellen	Anwendung
10	DiGAs sollten in der Lage sein, Daten aus dem Bestimmungszweck der DiGA und unter der Berücksichtigung des Datenschutzes aus anderen Quellen (z. B. „wearables“ oder andere Apps) zu integrieren	Datenflüsse
11	Gesundheits-Apps sollten nicht nur zur Therapie von Krankheiten bzw. deren Symptomen, sondern auch zur Prävention, Früherkennung und allgemein zur Förderung von gesundheitsförderndem Verhalten eingesetzt werden	Anwendung/Prävention
12	Clinical-Decision-Support-Systeme (CDSS) könnten ärztliches Personal bei der Entscheidungsfindung von Maßnahmen unterstützen	Arzt-App
13	Gesundheits-Apps könnten die Kommunikation zwischen Patient*innen und Gesundheitsdienstleistern erleichtern, indem sie sichere und einfache Kommunikationskanäle anbieten	AP-Kommunikation
14	Gesundheits-Apps könnten durch integrierte Übersetzungstools Sprachbarrieren überwinden, die im Gesundheitssystem zwischen den Patient*innen und Gesundheitsdienstleistern (z. B. Ärzte*innen/Pflege) bestehen könnten	AP-Kommunikation
15	Bei Gesundheits-Apps muss die Datensouveränität stets bei den Patient*innen liegen	Datensicherheit
16	Die Entwicklung von Gesundheits-Apps, auch an Hochschulen/Gesundheitseinrichtungen, sollte gesondert gefördert werden und nicht nur Herstellern überlassen werden	Förderung
17	Die aus DiGAs generierten Daten sollten anonymisiert für die wissenschaftliche Forschung zur Verfügung gestellt werden können	Forschung
18	Es sollten einheitliche Qualitätskriterien im europäischen Kontext zur Bewertung von Gesundheits-Apps implementiert werden	Qualitätskriterien
19	Gesundheits-Apps können die Effizienz des Gesundheitssystems durch eine Effizienzsteigerung/Qualitätsverbesserung der Kontakte zwischen den verschiedenen Akteuren (z. B. Patient*innen – Ärzt*innen) verbessern	AP-Kommunikation
20	Die Nutzung von Gesundheitsapps mit wissenschaftlicher Evidenz sollte nicht selektiv und aktiv von Krankenkassen beworben werden, um die Gesundheitskompetenz der Bevölkerung zu steigern	Förderung/Werbung
21	Eine nahtlose Integration von Daten aus DiGAs in die elektronische Patientenakte (ePA nach § 341 SGB V), wie in den DiGA-Zulassungskriterien gefordert, könnte eine bessere Übersicht schaffen und helfen sowohl dem behandelnden medizinischen Personal als auch den Patient*innen, fundierte Entscheidungen zu treffen	Datenflüsse
22	Es sollten Anreize für die Entwicklung von Gesundheits-Apps für Entwickler und Gesundheitseinrichtungen geschaffen werden	Förderung
23	Die Aus- und Weiterbildung von medizinischem Personal im Umgang mit digitalen Gesundheitslösungen sollte gefördert werden, um die Akzeptanz und den effizienten Einsatz von Gesundheits-Apps zu erhöhen	Ausbildung
24	Es fehlt eine Integration der DiGAs in technische Versorgungsstrukturen des deutschen Gesundheitswesens. Technisch sollten über Standardschnittstellen und durch die Vorgabe eines klaren rechtlichen Rahmens, Daten interoperabel leicht austauschbar sein	Datenflüsse/Integration in Gesundheitssystem
25	Zur vollumfänglichen Nutzung von Gesundheits-Apps kann der Einbezug von Wearables und deren Messwerten nützlich sein. Der entsprechende Zulassungsprozess sollte möglichst einfach gehalten werden	Zulassung
26	Agile Mechanismen zur kontinuierlichen Evaluation und Anpassung der gesetzlichen Rahmenbedingungen sollten geschaffen werden, um mit den schnellen technologischen Entwicklungen Schritt zu halten und Innovationen nicht zu behindern	Rechtliche Grundlage
27	Zulassungsprozesse müssen vereinfacht, transparent gestaltet und regulatorische Hürden in diesen Prozessen abgebaut werden	Zulassung
28	Die aus den DiGAs erhobenen Daten sollten nicht isoliert auf den mobilen Geräten oder beim Hersteller gespeichert werden. Zur langfristigen Speicherung sollte ausschließlich die elektronische Patientenakte verwendet werden	Datenflüsse/-speicherung
29	Von der Gesundheits-App erhobene Daten sollten nicht zum Nachteil der Patient*innen (z. B. durch die Kostenträger) genutzt werden können	Datensicherheit/keine Schädigung
30	Gesundheits-Apps mit wissenschaftlicher Evidenz sollten aufgrund ihres Nutzen-Risiko-Profils klassifiziert und die Anforderungen an Sicherheit, Evidenz und Nutzen und die Erstattung dementsprechend strukturiert werden	Klassifizierung von GAs

*DiGA* digitale Gesundheitsanwendung

### Anwendungsgebiete für Gesundheits-Apps mit chirurgischem Fokus

Entsprechend dem Konsens unserer Expertengruppe bieten Gesundheits-Apps Potenzial zur Unterstützung der chirurgischen Disziplinen. Sie können beispielsweise in der Patienteninformation eingesetzt werden (*Statement 3*). Zudem besteht Potenzial für die Erkennung, Überwachung oder Behandlung von Krankheiten sowie die Kompensation von Behinderungen (*Statement 1*). Gesundheits-Apps, die als DiGA zugelassen werden, durchlaufen ein strenges Prüfverfahren durch das Bundesinstitut für Arzneimittel und Medizinprodukte (BfArM), bevor sie als erstattungsfähige Leistung der gesetztlichen Krankenversicherung (GKV) verordnet werden können. Aktuell zugelassene DiGAs in Deutschland decken viele medizinische Bereiche ab, darunter auch Chirurgie oder innere Medizin, wobei der größte Anteil auf psychologische/psychiatrische Indikationen entfällt (*Statement 2*, Tab. 2). Für urologische Indikationen gibt es bereits wissenschaftliche Evidenz im Rahmen des Zulassungsverfahrens für DiGAs zu Ejaculatio praecox und Harninkontinenz [14, 15], 2 zugelassene DiGAs zur Behandlung der erektilen Dysfunktion und des Overactive-Bladder(OAB)-Syndroms/Benigne Prostatahyperplasie (BPS) sowie einer Therapie-App für Patienten nach radikaler Prostatektomie, die an Harninkontinenz und/oder erektiler Dysfunktion leiden [16] (*Statement 1*).

**Tab. 2:** Übersicht der chirurgischen DiGA (Stand: 18.10.2024) [9]

DIGA	Hersteller	Indikation/ICD Code	Beschreibung
Companion patella (dauerhaft aufgenommen)	PrehApp GmbH, Deutschland | prehapp.de	M22.2 Krankheiten im Patellofemoralbereich	Companion patella powered by medi – proved by Dt. Kniegesellschaft ist eine Digitale Gesundheitsanwendung für Patientinnen und Patienten mit vorderem Knieschmerz im Alter von 14 bis 65 Jahren. Basierend auf persönlichen Angaben des Anwenders zu Schmerz- und Belastungsempfinden wird der bewegungstherapeutische Trainingsplan im Verlauf der Therapie kontinuierlich an die individuellen Bedürfnisse der Nutzenden angepasst. Den Patient*innen steht neben dem Trainingsbereich eine Fachbibliothek mit Beiträgen zu ihrem Erkrankungsbild zur Verfügung, die diese edukativ unterstützt. Daten zu Schmerz und Funktionseinschränkungen werden innerhalb der Anwendung regelmäßig erfasst und für die Patient*innen und deren behandelnde Ärztinnen und Ärzte grafisch aufbereitet. Die Anwendungsdauer beträgt 90 Tage
		M22.4 Chondromalacia patellae	
		M76.5 Tendinitis der Patellarsehne	
		M79.66 Schmerzen in den Extremitäten: Unterschenkel [Fibula, Tibia, Kniegelenk]	
		S83.0 Luxation der Patella	
Kaia Rückenschmerzen – Rückentraining für zu Hause (dauerhaft aufgenommen)	kaia health software GmbH, Deutschland	M54 Rückenschmerzen	Kaia Rückenschmerzen ist eine digitale Anwendung für erwachsene Patient*innen mit nichtspezifischen Rückenschmerzen. Sie vermittelt leitlinienbasierte, an das Krankheitsstadium der Patient*innen angepasste Kerninhalte der multimodalen Therapie. Kaia Rückenschmerzen setzt sich aus den Therapieelementen Bewegung, Wissen und Entspannung zusammen
Kranus Edera (dauerhaft aufgenommen)	Kranus Health GmbH, Deutschland	N48.4 Impotenz organischen Ursprungs	Kranus Edera ist eine digitale Gesundheitsanwendung zur ganzheitlichen Behandlung von Erektionsstörungen und ihren Ursachen. Sie unterstützt Männer mit erektiler Dysfunktion, selbst aktiv an ihrer Behandlung teilzuhaben, und hilft Ärztinnen und Ärzten dabei, die Empfehlungen der Leitlinien zur Behandlung von Erektionsstörungen umzusetzen und so die Therapieoptionen zu erweitern. Anwender der App absolvieren ein 12-Wochen-Programm aus den Bausteinen Beckenbodentraining, physiotherapeutische Übungen, kardiovaskuläres Ausdauertraining sowie Achtsamkeits- und sexualtherapeutische Übungen. Patient*innen erhalten wöchentlich neue Übungen, die in ihrer Intensität und Komplexität fortlaufend angepasst werden. Ergänzt wird die Therapie durch Wissensvermittlung über die Erkrankung und hilfreiche Tipps, z. B. zur Ernährung und zu vorbeugenden Maßnahmen
Kranus Lutera (dauerhaft aufgenommen)	Kranus Health GmbH, Deutschland	N32.8 Sonstige näher bezeichnete Krankheiten der Harnblase	Kranus Lutera ist eine digitale Therapie für Männer mit Blasenentleerungsstörungen, auch „lower urinary tract symptoms“ (kurz „LUTS“) genannt. Kranus Lutera bietet eine einzigartige, einfache und personalisierte Therapie über 12 Wochen, die darauf abzielt, Männer mit häufigem Harndrang bei BPH und OAB durch eine Vielzahl von Funktionen und personalisierten Ansätzen zu unterstützen
		N40 Prostatahyperplasie	
Mawendo (dauerhaft aufgenommen)	Mawendo GmbH, Deutschland	M22 Krankheiten der Patella	Mawendo stellt Trainingsprogramme mit Übungsvideos, Gesundheitsinformationen und Dokumentationsmöglichkeiten bereit. Das Trainingsprogramm wird initial durch Ärztinnen und Ärzte ausgewählt und in Bezug auf das Krankheitsbild, die spezifischen Übungen sowie die Trainingsphasen für die Patient*innen individualisiert. Im Anschluss können Patient*innen mithilfe der DiGA die Behandlung von Erkrankungen der Kniescheibe (Patella) durch Eigentraining selbstständig unterstützen
Orthopy bei Knieverletzungen (vorläufig aufgenommen)	Orthopy Health GmbH, Deutschland	M23.2 Meniskusschädigung durch alten Riss oder alte Verletzung	Die Orthopy-App ist eine digitale Gesundheitsanwendung für Patient*innen mit einem Riss des vorderen Kreuzbandes und/oder Meniskusschädigungen. Die Orthopy-App begleitet Patient*innen vor, während und nach einer orthopädischen Behandlung. Orthopy unterstützt durch: verständliche Wissensbeiträge, physiotherapeutische Trainingspläne für das Heimtraining, leitliniengerechte Übungen und die Darstellung des sichtbaren Therapiefortschritts als Motivationshilfe
		M23.3 Sonstige Meniskusschädigungen	
		M23.61 Sonstige Spontanruptur eines oder mehrerer Bänder des Kniegelenkes: Vorderes Kreuzband	
		S83.2 Meniskusriss, akut	
		S83.53 Verstauchung und Zerrung des Kniegelenkes: Riss des vorderen Kreuzbandes	
PINK! Coach (dauerhaft aufgenommen)	PINK gegen Brustkrebs GmbH, Deutschland	C50 Bösartige Neubildung der Brustdrüse [Mamma]	PINK! Coach ist eine digitale Anwendung, die zur Stärkung der gesundheitsbezogenen Lebensqualität und der Gesundheitskompetenz sowie einer Linderung der psychischen, psychosomatischen und somatischen Folgen einer Brustkrebserkrankung dient. Um eine langfristige, anhaltende Verbesserung der Lebensqualität und des Gesundheitszustandes zu erreichen, zielt PINK! Coach darauf ab, das gesundheitsrelevante Verhalten der Patient*innen schrittweise, aber nachhaltig zu verändern
Selfapy-Online-Kurs bei chronischen Schmerzen (vorläufig aufgenommen)	Selfapy GmbH, Deutschland	F45.40 Anhaltende somatoforme Schmerzstörung	Der Selfapy-Online-Kurs ist eine digitale Anwendung für Betroffene von chronischen Schmerzen. Der Kurs vermittelt Methoden und Techniken, basierend auf der kognitiven Verhaltenstherapie, und unterstützt bei der Durchführung sowie der Dokumentation der Übungen mit dem Ziel, die Symptomatik der oder des Anwendenden zu verbessern
		F45.41 Chronische Schmerzstörung mit somatischen und psychischen Faktoren	
		M54 Rückenschmerzen	
Untire® (vorläufig aufgenommen)	Tired of Cancer B.V., Niederlande	C50 Bösartige Neubildung der Brustdrüse [Mamma]	Die Untire®-Applikation ist eine evidenzbasierte digitale Gesundheitsanwendung (DiGA), speziell entwickelt, um Erschöpfung (Fatigue) bei Brustkrebspatient*innen sowie Überlebenden zu reduzieren
ViViRA (dauerhaft aufgenommen)	Vivira Health Lab GmbH, Deutschland	Rückenschmerzen M42.0, M42.1, M42.9, M53.2, M53.8, M53.9, M54.4, M54.5, M54.6, M54.8, M54.9, M99.02, M99.03, M99.04, M99.8, M99.83, M99.84, M99.92, M99.93, M99.94	Die DiGA Vivira ist eine durch das BfArM endgültig ins DiGA-Verzeichnis aufgenommene digitale Anwendung zur Behandlung von Rückenschmerzen bei nichtspezifischen Kreuzschmerzen oder Arthrose der Wirbelsäule (Osteochondrose)
			Die bewegungstherapeutische DiGA Vivira bietet täglich 4 Übungen, die auf Basis der Rückmeldungen der Patient*innen fortlaufend ihre Intensität und Komplexität anpassen. Die täglichen Übungen werden durch wöchentliche Abfragen zur Gesundheit, die Visualisierung des Fortschritts, monatliche Bewegungstests und durch edukative Inhalte ergänzt. Vivira unterstützt die Umsetzung der in Leitlinien für nichtspezifischen Kreuzschmerz vorgesehenen Trainingselemente sowie die Umsetzung der Heilmittelrichtlinie

*ICD* International Classification of Diseases

Darüber hinaus können Gesundheits-Apps nicht nur therapeutisch, sondern auch präventiv eingesetzt werden. Sie spielen eine wichtige Rolle bei der Früherkennung und unterstützen gesundheitsbewusstes Verhalten (*Statement 11*). Diese Apps sollten einer breiten Patientengruppe ohne Einschränkungen bezüglich Alter oder finanzieller Mittel zugänglich sein, um chronische Erkrankungen zu überwachen und personalisierte Gesundheitspläne zu entwickeln (*Statement 5, 8, 9*).

Im chirurgischen Umfeld bieten „ärztliche Apps“ eine wertvolle Ergänzung zur Ausbildung und können helfen, chirurgische Fertigkeiten zu verbessern. Unsere Expertengruppe ist der Ansicht, dass diese Apps Ärzt*innen in Weiterbildung bei therapeutischen Anwendungen mobil unterstützen oder trainieren könnte (*Statement 6*). Zusammenfassend fördern DiGAs und Gesundheits-Apps nicht nur die Therapie, sondern auch gesundheitsbewusstes Verhalten und tragen zur Optimierung der Patientenversorgung bei. Sie haben das Potenzial, Versorgungslücken zu schließen und gleichzeitig Strukturen, Prozesse und Ergebnisse, insbesondere in der chirurgischen Versorgung, nachhaltig zu verbessern.

#### Regulatorik/Zulassungsprozesse

Eine DiGA muss ein zugelassenes Medizinprodukt sein, das zusätzliche Kriterien erfüllen muss, um von der gesetzlichen Krankenversicherung (GKV) erstattet zu werden. Diese Anforderungen werden im Leitfaden des BfArM erläutert, der bis Dezember 2023 bereits 10-mal aktualisiert wurde. Der Leitfaden ist jedoch zunehmend komplexer geworden, was nur bedingt den Forderungen unserer Konsensgruppe nach agilen Mechanismen zur Evaluation und Anpassung gesetzlicher Rahmenbedingungen entspricht (*Statement 26*).

Zusätzlich zu DiGAs existieren Gesundheits-Apps, die (noch) nicht als DiGA zugelassen, aber als Medizinprodukt klassifiziert sind, sowie zahlreiche Wellness-Apps (in der Regel keine Medizinprodukte). Eine App, die der „Diagnose, Verhütung, Überwachung, Vorhersage, Prognose, Behandlung oder Linderung von Krankheiten“ dient, gilt als Medizinprodukt [17].

Medizinprodukte einschließlich Gesundheits-Apps werden gemäß der EU-Verordnung in Risikoklassen eingeteilt. Hier fordert unsere Konsensgruppe eine Vereinfachung der Zulassungsprozesse und den Abbau regulatorischer Hürden (*Statement 27*). Aktuell neigt Europa dazu, medizinische Software in höhere Risikoklassen einzustufen als das Vereinigte Königreich oder die USA [18]. Eine öffentliche Konsultation der Medical-Device-Regulation-Vorschriften ist von der EU-Kommission geplant und könnte unsere Forderungen (*Statements 26, 27*) unterstützen [19]. Ziel ist eine klarere und vereinfachte Klassifizierung von Gesundheits-Apps.

Viele Gesundheits-Apps nutzen „Wearables“, um ihre Funktionen zu erweitern, was wir ausdrücklich begrüßen (*Statement 25*). Das seit dem 26.03.2024 geltende Digital-Gesetz vereinfacht die Integration von Wearables in DiGAs, da diese häufiger der Risikoklasse IIb zugeordnet werden, was auch für DiGAs zukünftig möglich sein soll.

Die strukturiert erhobenen Daten von Gesundheits-Apps sind wertvoll für die Forschung. Dies wird durch das in § 303d SGB V definierte „Forschungsdatenzentrum“ unterstützt. Allerdings wäre die Forderung nach wissenschaftlicher Nutzung anonymisierter DiGA-Daten (*Statement 17*) erst dann vollständig erfüllt, wenn die zusätzliche in § 363 (8) SGB V vorgesehene Möglichkeit, dass Versicherte ihre elektronischen Patientenakten-Daten mit Einwilligung für Forschung freigeben können, tatsächlich umgesetzt wird.

#### Evidenz/Qualitätskriterien

Gesundheits-Apps und DiGAs bieten großes Potenzial für die Modernisierung der chirurgischen Gesundheitsversorgung, stellen jedoch die digitale Gesundheitslandschaft vor Herausforderungen [3, 12, 20]. Damit DiGAs echten Mehrwert liefern und beispielsweise die Patientenautonomie fördern, müssen klare Evidenz- und Qualitätskriterien erfüllt sein [2, 21–24]. Der Konsens im Delphi-Verfahren betont, dass Nutzen und Sicherheit von Gesundheits-Apps in klinischen Studien überprüft werden müssen, um Wirksamkeit und Risikofreiheit sicherzustellen (*Statement 4*). Dies ist essenziell, da DiGAs oft bei chronischen Krankheiten oder psychischen Störungen eingesetzt werden. Einheitliche europäische Qualitätsstandards zur Bewertung von Gesundheits-Apps würden grenzüberschreitende Vergleichbarkeit, Verlässlichkeit und Vertrauen stärken (*Statement 18*).

Gesundheits-Apps können die Effizienz des Gesundheitssystems durch verbesserte Kontakte zwischen Akteuren wie Patient*innen und Ärzt*innen erheblich steigern (*Statement 19*). Shared Decision Making, also die gemeinsame Therapieentscheidung auf Basis von Evidenz und Patientenpräferenzen, kann ideal durch digitale Entscheidungshilfen unterstützt werden [25–27]. Zudem optimieren Gesundheits-Apps Diagnose- und Behandlungsprozesse durch präzisere Überwachung und Kommunikation.

Strenge Qualitätskriterien sind unerlässlich. Apps mit wissenschaftlicher Evidenz sollten nach Nutzen/Risiko klassifiziert und Anforderungen an Sicherheit, Nutzen und Erstattung entsprechend geregelt werden (*Statement 30*). Nur so können Mehrwert, Risikominimierung und Kosteneffizienz sichergestellt werden. Eine klare Regulierung stärkt das Vertrauen der Nutzer*innen und Ärzt*innen und verbessert langfristig die Versorgungsqualität.

#### Datenschutz und Datenhoheit

Im Rahmen der digitalen Gesundheitsversorgung durch Gesundheits-Apps sind Datenschutz und Datensouveränität essenziell. Patient*innen müssen jederzeit die Kontrolle über ihre Gesundheitsdaten behalten (*Statement 15*). Die Nutzung und Weitergabe der Daten ist verständlich und transparent darzustellen. Nutzer sollten die Einwilligung zur Datennutzung jederzeit widerrufen und gespeicherte Daten vollständig löschen können. Zulassungspflichtige DiGAs erfüllen bereits strikte Datenschutzanforderungen, die die Nutzung sensibler Daten nur im gesetzlichen Rahmen erlauben.

Gesundheits-Apps können die Kommunikation zwischen Patient*innen und Gesundheitsdienstleistern durch niederschwellige, datenschutzkonforme und verschlüsselte Kanäle erleichtern (*Statement 13*). Besonders vorteilhaft wäre, wenn die Daten nicht auf dem Endgerät der Patient*innen oder beim Hersteller gespeichert würden, sondern langfristig in die elektronische Patientenakte einfließen und so für Behandler zugänglich wären (*Statement 28*).

Wesentlich ist, dass Gesundheitsdaten nicht nachteilig gegen Patient*innen verwendet werden dürfen. Insbesondere Versicherungen und Krankenkassen dürfen Patientendaten nicht zur Erhöhung von Prämien oder Einschränkung von Leistungen nutzen, da dies das Vertrauen in Gesundheits-Apps beeinträchtigen könnte. Gesetzliche Maßnahmen müssen sicherstellen, dass Diskriminierung oder Benachteiligung aufgrund von Gesundheitsdaten ausgeschlossen wird. Gesundheits-Apps sollen ausschließlich der Verbesserung der Gesundheit und des Wohlbefindens dienen, nicht als Überwachungs- oder Diskriminierungsmittel genutzt werden (*Statement 29*).

#### Implementierung in die Praxis

Die Implementierung von Gesundheits-Apps in die Praxis bietet ein erhebliches Potenzial, um die Gesundheitsversorgung zu verbessern und die Gesundheitskompetenz der Bevölkerung zu fördern. Dabei ist es essenziell, dass die Entwicklung solcher Apps nicht ausschließlich kommerziellen Herstellern überlassen wird. Unsere Konsensusgruppe stellt fest, dass Hochschulen und Gesundheitseinrichtungen eine zentrale Rolle bei der Sicherstellung einer evidenzbasierten Entwicklung digitaler Gesundheitslösungen spielen sollen (*Statement 16*). Es ist belegt, dass die Einbindung dieser Institutionen zu einer besseren Qualität der Apps und zu einer höheren Patientenzentrierung führen kann [28]. Ein weiterer zentraler Aspekt der erfolgreichen Implementierung von Gesundheits-Apps ist die aktive Förderung durch Krankenkassen.

Digitale Gesundheitsanwendungen sind besonders wirksam in der Prävention und dem Management chronischer Erkrankungen, wenn sie nicht nur den bereits informierten Patient*innen angeboten werden, sondern auch aktiv durch Krankenkassen für deren Nutzung geworben wird [29] (*Statement 20*). Dies erhöht die Reichweite und spricht einen größeren Teil der Bevölkerung an. Durch die Bewerbung von Apps, die auf wissenschaftlicher Evidenz beruhen, kann zudem die Gesundheitskompetenz in der Bevölkerung langfristig gesteigert werden [6]. Anreize für Entwickler und Gesundheitseinrichtungen sind ebenfalls wichtig, um die Weiterentwicklung von Gesundheits-Apps voranzutreiben. Die Konsensusgruppe ist sich einig, dass solche Anreize in Form von finanziellen Unterstützungen, Innovationsförderungen oder steuerlichen Vorteilen angeboten werden sollten [7] (*Statement 22*). Dies stellt sicher, dass die Qualität und Innovationskraft der Apps erhalten bleiben und sie einem breiten Nutzerkreis zugänglich sind. Insgesamt ist die Schaffung eines förderlichen Umfelds für die Entwicklung, Bewerbung und Nutzung von Gesundheits-Apps ein wichtiger Schritt hin zu einer modernen, digitalisierten Gesundheitsversorgung.

#### Interoperabilität und technische Schnittstellen

Die Integration von mobilen Gesundheits-Apps in die Krankenhausarbeitssysteme hat das Potenzial, die Effizienz von Dokumentationsprozessen zu steigern und Arbeitsabläufe zu optimieren. Die Dokumentation von Patientendaten in Krankenhäusern ist häufig zeitaufwendig und fehleranfällig, was die Arbeitsbelastung des Personals erhöht und die Patientenversorgung beeinträchtigen kann. Der Einsatz von Apps könnte diese Prozesse durch Echtzeiterfassung und zentralisierte Speicherung der Daten vereinfachen und beschleunigen. Studien zeigen, dass digitale Dokumentationssysteme die Genauigkeit der Datenerfassung verbessern und den Zeitaufwand für administrative Tätigkeiten verringern können [30]. Dies entlastet das medizinische Personal und steigert die Effizienz in Krankenhäusern (*Statement 7*).

DiGAs spielen eine wachsende Rolle bei der Überwachung und Therapie von Patient*innen. Idealerweise sollten sie nicht nur eigenständige Daten erheben, sondern auch Daten aus anderen Quellen integrieren. Wearables und andere Gesundheits-Apps bieten eine Fülle von Daten, die durch ihre Verknüpfung mit DiGAs zu personalisierten und genaueren Therapieentscheidungen führen könnten. Besonders bei chronischen Erkrankungen wie Diabetes oder Herz-Kreislauf-Erkrankungen ermöglicht die Integration solcher Daten eine kontinuierliche Überwachung und Anpassung der Therapie [31]. Dies stellt jedoch eine Herausforderung bezüglich der Zertifizierungsauflagen dar, und es muss auf die strikte Einhaltung der Datenschutzrichtlinien geachtet werden, um die Sicherheit und Vertraulichkeit der Patientendaten zu gewährleisten (*Statement 10*).

Gesundheits-Apps mit integrierten Übersetzungstools könnten die Inklusion von Patientengruppen fördern, die aufgrund sprachlicher Barrieren schlechter versorgt werden. Dies ist besonders in Notfällen von Bedeutung, in denen schnelle und präzise Kommunikation entscheidend sein kann. Studien zeigen, dass Übersetzungs-Apps in Notaufnahmen das Vertrauen der Patient*innen erhöhen und die Behandlungsqualität verbessern können [32]. Die Integration solcher Tools in standardisierte Gesundheits-Apps könnte das Gesundheitssystem insgesamt effizienter und zugänglicher machen (*Statement 14*).

Ein klarer rechtlicher Rahmen und verbindliche Standards sind nötig, um sicherzustellen, dass DiGA-Daten sicher und effizient zwischen den Akteuren im Gesundheitssystem ausgetauscht werden können [11] (*Statement 24*). Der Bundestag und die Gesellschaft für Telematikanwendungen der Gesundheitskarte mbH (Gematik) betonen diesen Schritt zur Verbesserung der digitalen Gesundheitsversorgung [33, 34]. Eine Integration der DiGA-Daten in die elektronische Patientenakte (ePA nach § 341 SGB V) könnte sowohl dem medizinischen Personal als auch den Patient*innen helfen, fundierte Entscheidungen zu treffen und die Behandlungsqualität zu verbessern (*Statement 21*). Dies stärkt die Eigenverantwortung und Patientensicherheit, indem Patient*innen besser in den Entscheidungsprozess eingebunden werden.

#### Schulung und Ausbildung

Um die Nutzung von Gesundheits-Apps im Alltag zu fördern, ist die Weiterbildung des medizinischen Personals entscheidend. Schulungen stärken das Vertrauen der Anwender in digitale Lösungen und fördern deren effizienten Einsatz im klinischen Umfeld (*Statement 23*). Untersuchungen belegen, dass solche Programme die Akzeptanz und Anwendung digitaler Tools erhöhen. Gesundheits-Apps können den medizinischen Ausbildungsprozess unterstützen und optimieren, insbesondere durch die Förderung spezifischer Fähigkeiten in der ärztlichen und chirurgischen Ausbildung. Sie können praktische Szenarien simulieren, um Fertigkeiten zu verbessern und die Qualität therapeutischer Anwendungen zu steigern (*Statement 6*). Clinical-Decision-Support-Systeme (CDSS) bieten wertvolle Unterstützung, indem sie medizinische Entscheidungen durch Zugang zu aktuellen Behandlungsrichtlinien und patientenspezifischen Daten optimieren (*Statement 12*). Studien zeigen, dass CDSS die Behandlungsqualität und Patientensicherheit verbessern können, indem sie evidenzbasierte Empfehlungen liefern [35].

## Schlussfolgerungen

Die Mitglieder der Konsensuskonferenz empfehlen dem Gesetzgeber, die Entwicklung und Implementierung von Gesundheits-Apps und DiGAs aktiv zu fördern. Die erarbeiteten 30 zentralen Statements verdeutlichen das Potenzial dieser Technologien, die Patientenversorgung in den chirurgischen Disziplinen erheblich zu verbessern, und formulieren Forderungen für die Anpassung von gesetzlichen Grundlagen sowie Förderung von Forschung und Weiterentwicklung auf diesem Gebiet.

Aus dem Bereich „Realität“ wurde festgehalten, dass Gesundheits-Apps einen evidenzbasierten Nutzen in der Patienteninformation haben können und unabhängig vom Alter der Patient*innen angeboten werden sollten. Sie können Symptome und Beschwerden von Krankheiten und krankhaften Zuständen reduzieren, personalisierte Gesundheitspläne, basierend auf individuellen Gesundheitsdaten, erstellen und die Überwachung und Therapie chronischer Erkrankungen unterstützen. Gesundheits-Apps können die ärztliche/chirurgische Ausbildung unterstützen und therapeutische Anwendungen verbessern sowie den Dokumentationsaufwand und die Prozessabläufe in Gesundheitseinrichtungen effizienter gestalten und Daten aus anderen Quellen (z. B. Wearables oder andere Apps) integrieren.

Aus dem Bereich „Utopie“ ergab sich der Konsens, dass Gesundheits-Apps zur Effizienzsteigerung und Qualitätsverbesserung der Kontakte zwischen den verschiedenen Akteuren (z. B. Patient*innen – Ärzt*innen) beitragen können sowie Sprachbarrieren durch Übersetzungstools überwinden. Es gilt, einheitliche Qualitätskriterien im europäischen Kontext zur Bewertung von Gesundheits-Apps zu entwickeln. Die Datensouveränität muss stets bei den Patient*innen liegen, und Daten sollten anonymisiert für die wissenschaftliche Forschung zur Verfügung stehen. Die Entwicklung von Gesundheits-Apps sollte auch an Hochschulen/Gesundheitseinrichtungen vorgenommen werden und die Nutzung nicht nur selektiv und aktiv von Krankenkassen beworben werden.

Aus dem Bereich „Transformation“ ließ sich feststellen, dass es zukünftig erforderlich sein wird, regulatorische Hürden abzubauen und dabei DiGAs auf Basis von wissenschaftlicher Evidenz und Nutzen-Risiko-Profilen zu klassifizieren. Daten aus DiGAs sollten in die elektronische Patientenakte integriert und erhobene Daten nicht zum Nachteil der Patient*innen (z. B. durch die Kostenträger) genutzt werden. Anreize für die Entwicklung von Gesundheits-Apps für Entwickler und Gesundheitseinrichtungen sollten gesetzt werden. Die Aus- und Weiterbildung von medizinischem Personal im Umgang mit DiGAs gilt es zu fördern und die Angebote in technische Versorgungsstrukturen zu integrieren.

Um das Vertrauen der Nutzer zu stärken, sind einheitliche Qualitätskriterien und umfassende Datenschutzrichtlinien unerlässlich. Die Kontrolle über Gesundheitsdaten muss für Patient*innen jederzeit gewährleistet sein, sodass sie jederzeit darüber entscheiden können, wie ihre Daten verwendet werden. Die Integration von Gesundheits-Apps in bestehende Gesundheitssysteme erfordert ein koordiniertes Handeln von Gesetzgeber, Hochschulen, Krankenkassen, Gesundheitsdienstleistern und App-Anbietern. Ziel sollte es sein, einen sicheren Austausch von DiGA-Daten zwischen verschiedenen Akteuren im Gesundheitswesen zu ermöglichen. Hierfür ist es wichtig, einen klaren rechtlichen Rahmen zu schaffen. Zusammenfassend appellieren wir an den Gesetzgeber, ein unterstützendes Umfeld zu schaffen, das die Entwicklung, Zulassung und Nutzung von Gesundheits-Apps gezielt und niederschwellig v. a. auch für akademische Gruppen fördert. Diese Maßnahmen sind entscheidend, um die Qualität der Gesundheitsversorgung zu verbessern und den Herausforderungen der modernen Gesundheitsversorgung gerecht zu werden.

## Supplementary Information

Zusatzmaterial online

## Data Availability

Auf begründeten Antrag können die Ergebnisse der einzelnen Delphi-Abstimmungsrunden über den entsprechenden Autor zur Verfügung gestellt werden.
