# Supplementary material for: Implementation and benefits of healthcare apps in surgical disciplines—A Delphi expert consensus
Source: Chirurgie (Heidelb). 2025 Jul 7;97(2):132–42. [Article in German] doi: 10.1007/s00104-025-02329-5 (PMC12864361; doi:10.1007/s00104-025-02329-5)
Supplement: Supplementary file 1 — Zusatzmaterial online [file 104_2025_2329_MOESM1_ESM.pdf]

Zusätzliche Tabelle 1: Auflistung der Mitglieder der Expertengruppe.

Name	Affiliation	Expertise
PD Dr. med. C. Groeben	Klinik für Urologie, Philipps-Universität Marburg	Urologe und Androloge, Wiss. Leiter der „CLIMACS-Studie“ zur Erforschung des Effekts einer Gesundheits-App auf die Symptome der Ejaculatio praecox); Wissenschaftl. Durchführung der Event-PCa-Studie (Online-Entshceidungshilfe für Patienten mit lokal begrenztem Prostatakarzinom).
Prof. Dr. psych. D. Ebert	School for Medicine & Health, Technische Universität München	Psychologe & Digital Health Forscher, >50 klinische Studien zu digitalen Gesundheitsinterventionen, Gründer & CSO HelloBetter, DGPS & DGPPN Taskforce eHealth, ehemals President-elect International Society for Research on Internet Interventions.
Dr. phil. P. Karschuck	Urologische Universitätsklinik Heidelberg	Klinikkoordinator und Wissenschaftlicher Mitarbeiter, Forschungsschwerpunkt: Medizinethik (Patienten-Empowerment, Entscheidungshilfe Prostatakrebs, Selbsthilfe).
Dr. med. L. Wiemer	Kranus Health Gmbh, München<br><br>Klinik für Urologie Charité, Berlin	Urologin und Expertin für digitale Gesundheitsanwendungen, Fachärztin in urologischer Praxis, externe wissenschaftliche Mitarbeiterin Charité und Chief Scientific Officer Kranus Health, Entwicklung von drei digitalen Gesundheitsanwendungen und Konzeption mehrerer RCTs und Studien im Bereich Digital Health.
Prof. Dr. med. A. Wiedemann	Klinik für Urologie, Ev. Krankenhaus Witten gGmbH<br><br>Universität Witten/ Herdecke	Urologe, Androloge, med. Tumortherapie; Chefarzt der Klinik für Urologie d. Ev. Krankenhaus Witten gGmbH und Lehrstuhlinhaber für Geriatrie d. Universität Witten/Herdecke. Leiter d. AK geriatrische Urologie d. DGU; Leiter AG Inkontinenz DGG; 1. Vorsitzender Dt. Kontinenz Gesellschaft; Prinzipal Investigator der Studie INKA Sys. Eval.
Dr. med. H. Krause	Universitätsklinikum Mannheim, INSPIRE Living Lab	Projektmanagerin des INSPIRE Living Lab. Das Reallabor bietet Startups und Firmen die Möglichkeit, MedTech- und Digital Health Produkte im klinischen Alltag in Kooperation mit Ärzt:innen, Pflege und Patient:innen zu testen und weiterzuentwickeln.
Dr. med. K. Fuchs, M. Sc.	Klinik und Poliklinik für Unfall-, Hand-, Plastische und Wiederherstellungs-chirurgie, Universitäts-klinikum Würzburg	Unfallchirurg, M. Sc. in Biomedizinischer Informatik und Data Science, Projektmanager der AG Digitalisierung der Universitätsmedizin Bayern.

PD Dr. med. M. Baunacke	Klinik und Poliklinik für Urologie, Universitäts-klinikum Dresden, TU Dresden	Urologe, Versorgungsforschung und funktionelle Urologie, Studienleiter der ProKontinenz-Studie (GBA-geförderte Studie für ein digitales Informationsprojekt).
Prof. Dr.med. A. Schnitzbauer	Klinik für Chirurgie am Knappschaftskranken-haus Langendreer, Ruhr-Universität, Bochum	Viszeralchirurg; Direktor der Abteilung für Experimentelle Chirurgie am Zentrum für Klinische Forschung der Ruhr-Universität sowie der Chirurgischen Klinik. Forschungsschwerpunkt Digitale und KI-gestützte Vorbereitung von Patienten auf onkologische Eingriffe (Prähabilitation). Studienleiter der LUMOS-Studie.
A. Schmitz, MBA	Bayer AG, Pharmaceuticals Division, Research and Development, Berlin	Naturwissenschaftler, Spezialisierung Health Care Administration und Patienten Apps; langjährige Betreuung und Mentoring von startups; hauptberuflich ‚Medical Software Product Lead'. Forschung mit ePROs, real world evidence Studien, QMS für Medizinprodukte.
